# Prevalence of sensitization to food allergens and challenge proven food allergy in patients visiting allergy centers in Rawalpindi and Islamabad, Pakistan

**DOI:** 10.1186/s40064-016-2980-0

**Published:** 2016-08-11

**Authors:** Muhammad Inam, Rubaba Hamid Shafique, Nabila Roohi, Muhammad Irfan, Shahid Abbas, Muhammad Ismail

**Affiliations:** 1 Physiology/Endocrinology Lab, Department of Zoology, University of the Punjab, Quaid-i-Azam Campus, Lahore, 54590 Pakistan; 2 Department of Zoology, University of Arid Agriculture Rawalpindi (PMAS-UAAR), Rawalpindi, Pakistan; 3 Allergy Asthma and Immunology Centre, Khyber Plaza Fazal-e-Haq Road, Blue Area, Islamabad, Pakistan; 4 Institute of Biomedical and Genetic Engineering (IBGE), Islamabad, Pakistan

**Keywords:** Food allergy, Skin Prick Test, Oral food challenge, Food sensitization

## Abstract

In this study, we estimated the prevalence of food allergy in the adult allergic patients of Rawalpindi and Islamabad , Pakistan, based on self-report, skin prick test (SPT) and oral food challenge test (OFC). SPT was used for the estimation of sensitization to wheat, egg, milk, beef, chicken, mutton, fish, corn, lentils, rice, soya, peanut and banana. Among 689 patients, 39.19 % showed sensitivity to one or more foods, where, sensitization to wheat (156; 22.6 %) was highest, followed by egg (148; 21.48 %) and milk (138; 20.03 %). Sensitization to various proteins ranged between 15.53–15.97 %, while lentils, corn, rice, soya and peanut sensitization was 15.4, 16, 12.5, 12 and 11.5 % respectively. Only 7.1 % patients were SPT positive for banana allergen. SPT was performed in patients with self-reported food allergy (341/689) and also with no self-reported history of food allergy (348/689). SPT results were positive in 69.8 % of the self-report group, whereas, in the patients with no self-reported food allergy 9.2 % were found sensitized to one or more tested food allergens. 101 patients were recruited for OFC, 61 % of these were confirmed of food allergy. The prevalence of food allergy in the study population was 9 %. Food specific OFC results show that wheat allergy is affecting 1.6 % (95 % CI 0.9–2.84 %) of the total allergy patients, followed by egg allergy 1.31 % (95 % CI 0.70–2.47 %). Furthermore, corn allergy, rice allergy and peanut allergy were 1.02, 0.87 and 0.73 %, respectively. In conclusion, wheat allergy is the most prevalent, followed by egg, chicken, beef and fish allergy, respectively.

## Background

Food allergy is a malfunction of the immune system in response to dietary antigens (Beyer and Teuber 2004). It may involve mechanisms that are IgE-mediated, non-IgE-mediated, or both (Garcia and Lizaso 2011), while all other non-allergic food reactions are categorized as “food intolerance”. Like all atopic allergies, food allergy develops in genetically predisposed individuals as a consequence of oral tolerance failure (Sampson 2004). Symptoms of food allergy are as diverse as in other allergies involving the oral cavity, skin, respiratory system, circulatory system and gastrointestinal tract. In case of acute food allergy generalized anaphylaxis may also occur (Ballmer-Weber et al. 2007; Kumar et al. 2010; Mackie et al. 2012).

Although there is no substantial scientific evidence, it is estimated that the prevalence of food allergy has increased over the past few decades (van Ree et al. 2015), reaching 3–6 % (exceeding 10 % in some regions), significantly influencing the quality of life and adding to the economic burden of a society (Hadley 2006). Changing dietary habits and availability of all kinds of food products around the world may be a cause of the increase in food allergies. An increase in the prevalence of some food allergies like peanut allergy (Sicherer et al. 2003) is not dependent on their increased consumption, but researchers believe that, changes in food production and processing may affect the allergenicity of these foods (Beyer et al. 2001; Chung et al. 2003; Schmitt and Maleki 2003).

The study of epidemiology of food allergies is important in order to elucidate the full gravity of this problem and establish a possible link between increased prevalence of asthma and allergic rhinitis with the rise in food allergy in the past decade (Prescott and Allen 2011). Epidemiological studies and prevalence data on food allergy are either based on self-reported allergic reactions or clinically based allergy tests. Despite reports of increasing prevalence of food allergies and food-induced anaphylaxis in the last decade, there is a need to standardize the methodologies used in various surveys to make a comparison of prevalence studies more objective (van Ree et al. 2015). Age, geographic location, and race/ethnicity are some of the additional factors influencing the prevalence of food allergy in a given population (Hadley 2006). For effective and unbiased determination of the food allergy occurrence, scientists feel the need to overcome misclassifications due to subjective participation in a survey, unavailability of simple diagnostic tests against all types of food allergens, rapidly evolving disease, increasing numbers of potential allergy causing foods, and variable clinical symptoms (Hadley 2006).

A confirmed diagnosis of food allergy necessitates a three dimensional approach. The first step is to identify the possibility of food allergy, based on patients’ history (adverse reaction after consumption of a particular food). The second step is the detection of food allergen specific IgE antibodies in the blood through Skin Prick Test (SPT) or immunoassay and finally to establish a clinical relevance of previous procedures with a positive food challenge [(Oral food challenge (OFC) or double-blind, placebo-controlled food challenge (DBPCFC)]. Clinicians usually omit confirmatory food challenges and declare positive food allergy when dealing with patients who have recently suffered from an anaphylactic attack from a particular food and are fulfilling other diagnostic criteria (Steckelbroeck et al. 2008). This procedure helps reduce self-report bias and gives a more objective estimate of food allergy prevalence in a study group (van Ree et al. 2015).

Allergy causing foods contain chemical substances (typically proteins, sometimes with small molecules called haptens) that elicit specific immunologic reactions causing hypersensitivity reactions if eaten uncooked, cooked or sometimes even after they have undergone digestion (Sicherer and Sampson 2010; Waserman and Watson 2011). Out of hundreds of different foods that humans consume, only a small number, account for most of the food allergic reactions. In children approximately 90 % of hypersensitivity reactions are caused by milk, eggs, peanuts, soybeans and wheat, whereas, in adults, fish, shellfish, tree nuts and peanuts account for approximately 85 % of allergic reactions (Krishna 2001). Up to 8 % of children and 2 % of adults in westernized countries suffer from allergic reactions against various foods (Beyer and Teuber 2004). Nearly 4 % of Americans (approximately 12 million) are affected by food allergies, including 3.7 % adults, 6 % children younger than 3 years of age (Sicherer and Sampson 2006). Children with atopic disorders tend to have a higher prevalence of food allergy; about 35 % of children with moderate to severe atopic dermatitis have IgE-mediated food allergy (Eigenann et al. 1998) and about 6 % of children with asthma have food-induced wheezing (Novembre et al. 1988).The National Institute of Allergy and Infectious Disease (NIAID-US) (Boyce et al. 2010) recently concluded that the prevalence of food allergy among all age groups lies between 1–10 % (Hefle et al. 1996; Sampson et al. 2005).

In the present study, we estimated for the first time, the prevalence of overall food allergy in adult patients visiting various allergy centers in Rawalpindi and Islamabad, Pakistan. The confirmation of allergy was based on the three steps’ standard approach. At the same time SPT followed by an OFC test was used to establish the status of sensitization to various food types viz. wheat, egg, milk, beef, chicken, mutton, fish, corn, lentils, rice, soya, peanut and banana.

## Methods

**Fig. 1:**
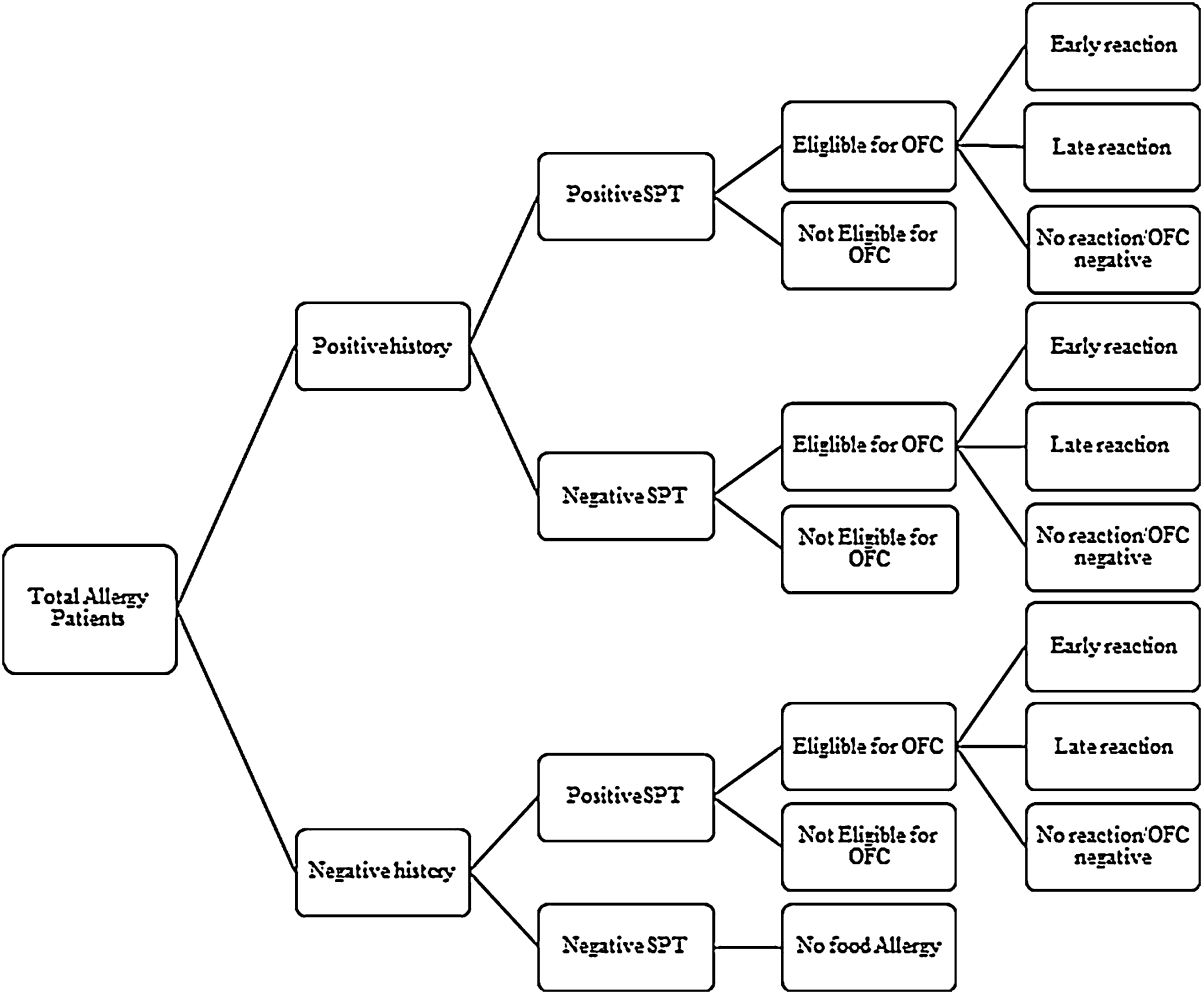
Methodology used in the present study

The participants were recruited after an informed consent and approval of the concerned authorities. Patients visiting the various allergy centers were divided into two groups: patients with positive food reaction history and negative food reaction history. Each group underwent Skin Prick Test (SPT) and was further categorized as positive SPT/negative SPT. All positive SPT patients were assessed for their eligibility to undertake the OFC test. The reactions observed were designated as early, late and no reaction according to the time taken for the symptoms to appear (Fig. 1).

### Skin Prick Test (SPT)

Allergen extracts for SPT were purchased from Hollister-Steir (Spokane, WA). Keeping in view the nutritional habit of Pakistani population, 13 food allergens, including milk, beef, chicken, egg, mutton, fish, banana, lentils, peanut, rice, soya corn and wheat were used, crustacean seafood is not consumed as diet in local population therefore, it was not considered for test. Volar aspect of forearm 2–3 cm from the wrist of the patient was cleaned with ethanol and marked for individual allergens. The distance between skin marks was at least 2 cm to avoid false positives. Allergen solution was placed beside each mark and the skin was pricked with a sterile lancet. The excess allergen solution was dabbed off. After 20–30 min’ appearance of a flare (red inflammation/wheel) was noticed and its size was measured. Wheel size ≥3 mm was considered as positive (Heinzering et al. 2013; Konstantinou et al. 2009).

### Diagnostic oral food challenge (OFC)

Food allergy was diagnosed through oral food challenge at the allergy center under the supervision of a trained physician. Prior to OFC, patients’ history was reviewed, each individual was physically examined and baseline of vital signs i.e. spirometry, blood pressure and heart rate etc. were recorded. Patients having recent anaphylaxis, severe asthmatic attack, cardiovascular disease, using beta blockers and carrying pregnancy were eliminated from the OFC group. An informed consent was obtained from each patient who recruited for OFC. Food suspected for allergy was strictly eliminated from the diet of recruited individuals for 2 weeks prior to an OFC and lastly, medications i.e. H1 antihistamines (oral/intranasal), H2 antihistamine, antidepressants and steroids etc. that may interfere with interpretation were also discontinued 72 h before the test.

Oral food and fluids were discontinued at least 12 h prior to OFC. During the OFC procedure, patients were monitored/supervised and regularly re-examined prior to each dose and at first signs of reaction. Spirometry was repeated if respiratory symptoms were observed. Total dose was divided into 6 incremental portions, where every next dose was double of the previous dose e.g. 1, 2, 4, 8 and 16 g of a solid food or 1, 2, 4, 8, 16 ml of liquid food. In case of suspected severe reaction a much smaller dose was decided. A few randomly selected doses in each test, were disguised to include blinded OFC and reduce any bias from the challenge.

In cases where patients’ complained of subjective symptoms (symptoms complained by the patients, i.e. throat or mouth itching, skin itching, or nausea) an observation period was allowed for the development of observable signs of an allergic reaction. However, in case no observable indications developed, OFC was continued. The test was considered positive if subjective symptoms followed 3 doses of the test food, with a 30-min interval between each dose (Niggemann 2010; Ito and Urisu 2009). OFC was continued, in spite of the subjective symptoms, until the subject exhibited objective symptoms (i.e. occurrence of flush, local urticaria, or a slight worsening of an underlying eczema). However, in a few cases the challenge was discontinued due to uneasiness or unwillingness of patients to continue the challenge (Niggemann 2010).

The results were considered “positive” until ‘moderate to severe objective clinical reactions’ occurred, such as respiratory, gastrointestinal, skin and cardiovascular symptoms (Ito and Urisu 2009). The challenge was stopped as soon as the observer(s) was/were convinced that a reaction is occurring. All medications that were needed before the challenge and stopped for OFC were administered without delay. OFC was considered negative when the patient tolerated the entire challenge, including the masked and open portions of the OFC. In case of a negative OFC patient was discharged after an observation period of ≥2 h. In case of a positive OFC, the patients were retained under observation for 2–4 h after resolution of symptoms (with or without treatment) depending upon the severity (mild or minimal residual) of the reaction. At the time of discharge from allergy center they were instructed to keep a log of symptoms for at least 48 h and report back in the clinic in case of late reaction. Patients with a past history of severe reactions, were kept under observation for a long period (≥6 h) even though their symptoms were completely resolved. Skin (oedema, facial flushing, rash or hives) and respiratory symptoms (fall in peak expiratory flow rate or PEFR, severe wheezing) were the most frequent, while a few suffered from gastrointestinal symptoms (vomiting and diarrhea) (Ito and Urisu 2009).

## Results

### Sensitization to food allergens

**Fig. 2:**
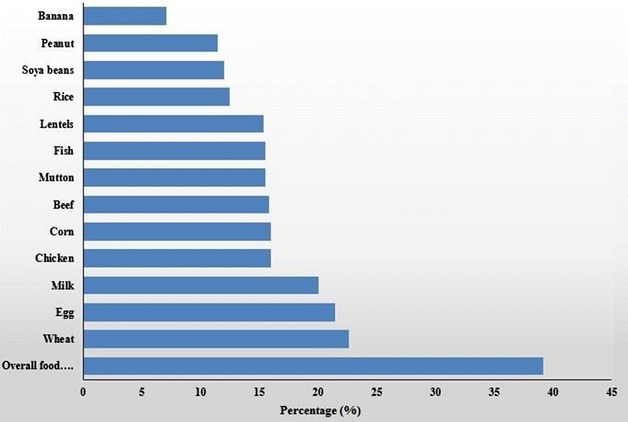
Sensitization (%) to various food allergens in the study group (n = 689)

Overall 689 patients were registered in the study. Their demographic characteristics are shown in Table 1. Two hundred and seventy (277; 39.2 %) patients were sensitized to one or more food allergens, wherein, 266 (98.5 %) patients were poly-sensitized giving positive SPT to more than one tested food allergen. On the other-hand 419 (60.8 %) individuals in the study were SPT positive to other types (i.e. HDM, pollen, dust, etc.) of allergens. Sensitization to wheat (156; 22.6 %) was the most frequent among the food allergens tested followed by sensitization to egg, i.e. 148; 21.5 % (Table 1; Fig. 2). Sensitization to milk was observed in 138 (20 %) patients, whereas, sensitization to proteins from various meat sources ranged between 15.5–16 %. SPT for lentils, corn, rice, soya and peanut was positive in 106 (15.4 %), 110 (16 %), 86 (12.5 %), 83 (12 %) and 79 (11.5 %) patients respectively. Only 49 (7.1 %) patients were SPT positive for banana allergen (Fig. 2).

**Table 1:** Demographic characteristics of the participants

Characteristics		Patients N (%)	95 % (CI)
Age	Range (median)	n = 689	
	15–73 (36) years		
Gender			
F		302 (44)	40.17–47.56
M		387 (56)	52.44–59.83
Sensitization to food allergens		270 (39)	35.61–42.89
Animal proteins			
Milk		138 (20)	17.2–23.18
Beef		109 (16)	13.29–18.73
Chicken		110 (16)	13.42–18.89
Egg		148 (21)	18.58–24.7
Mutton		107 (16)	13.02–18.42
Fish		107 (16)	13.02–18.42
Plant foods			
Banana		49 (7)	5.42–9.28
Lentils		106 (15)	12.88–18.27
Peanut		79 (12)	9.33–14.10
Soya		83 (12)	9.83–14.69
Rice		86 (13)	10.22–15.16
Corn		110 (16)	13.42–18.89
Wheat		156 (3)	19.67–25.91
Other allergens			
HDM, pollen, dust etc.		419 (61)	57.11–64.39
Symptoms			
Respiratory		522 (76)	72.42–78.81
Cutaneous		250 (36)	32.78–39.94
OFC reaction		*n* *=* *101*	
Early reaction (within 2 h)		50 (50)	39.95–59.09
Late reaction (2–48 h)		12 (12)	6.93–19.63
Negative (after 48 h)		39 (39)	29.7–48.36
Treatment			
Yes		265 (38)	34.90–42.51
No		424 (62)	57.85–65.10

**Fig. 3:**
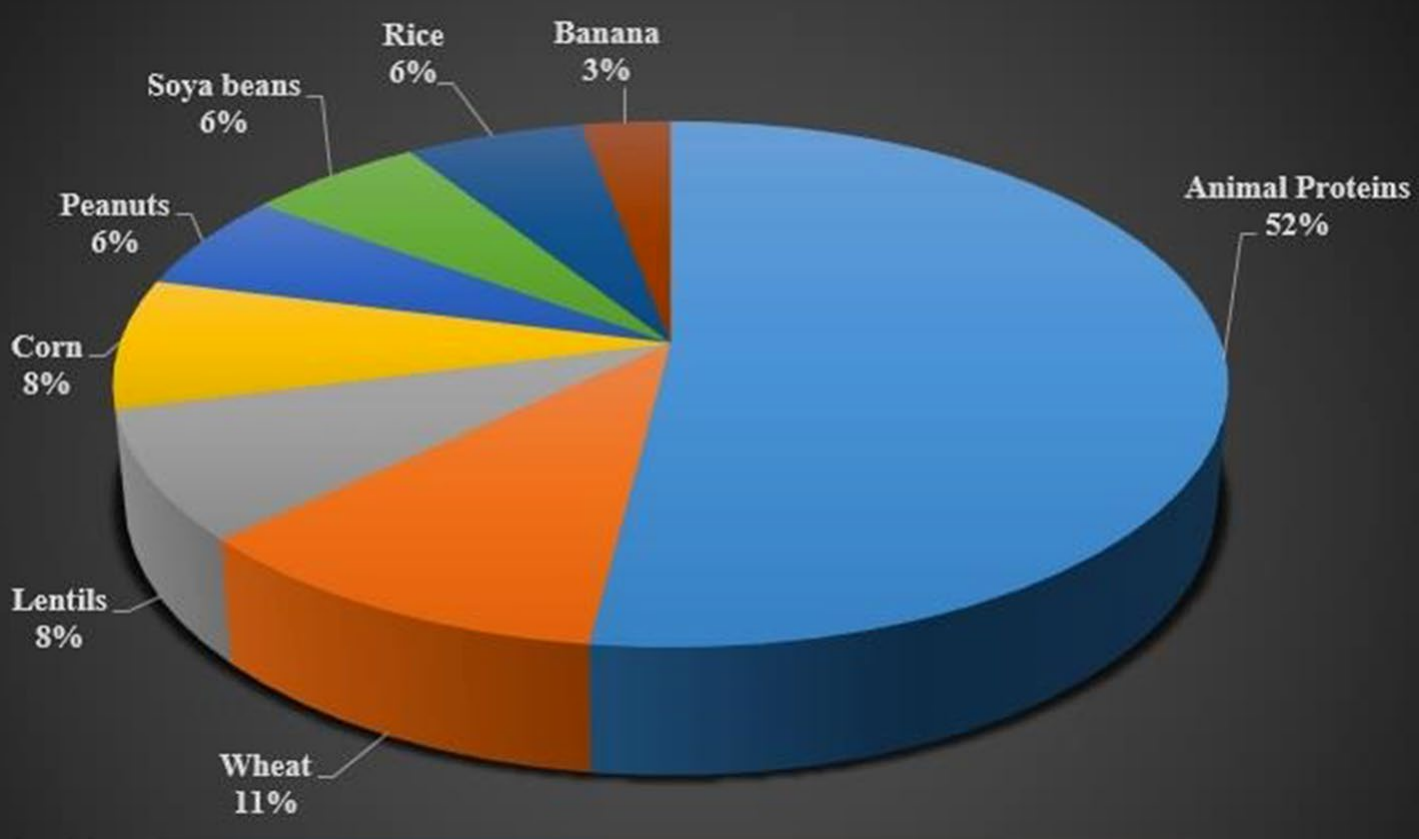
Distribution of percent sensitization to various food allergens in SPT positive group (n = 270)

Among 270 food sensitized individuals, animal protein (e.g. milk, beef, chicken, egg, mutton or fish) sensitivity was the most common (47 % prevalence) followed by wheat sensitivity (10 %) (Fig. 3).

### OFC based food allergy

Out of 689 adult patients (age 15–73 years) that visited the allergy clinic, 341 (49.5 %) had self-reported history of food allergy whereas 348 (50.5 %) did not report any adverse food reactions. All patients were screened through SPT to determine food sensitivity. Two hundred and thirty-eight (238) out of 341 (69.8 %) self-reported allergy patients were SPT positive to 1 or more tested allergens, whereas 103 (30.2 %) did not show sensitization to any food allergen (Table 2). Among 348 individuals who did not experience any food allergy symptoms, 32 (9.2 %) were found sensitized to one or more tested food allergens while 316 (90.8 %) presented a negative SPT. These patients were not included in open OFC and were designated negative for food allergy. Patients from all the remaining groups were assessed for their eligibility to undertake the open OFC test. Sixty-one percent (61 %: 62/101) of the patients that participated in OFC were diagnosed of food allergy. In the total study population (n = 689) the prevalence of food allergy was 9 %.

**Table 2:** An overview of participation in the oral food challenge (OFC)

Total allergy patients (n = 689)	Patient history (N)	Skin Prick Test (SPT) results (N)	Eligible for OFC (N)	Not eligible for OFC (N)
Positive	341	Positive (238)	54	184 [An (9), SA (98), CvP (34), CP (20) and R (23)]
		Negative (103)	36	67
Negative	348	Positive (32)	11	21 [SA (2), CvP (9), CP (3) and R (7)]
		Negative (316)	No food allergy	

*An* anaphylaxis; *SA* severe asthma; *CvP* cardiovascular patients/β-blocker users; *CP* carrying pregnancy; *R* refusal

In the open OFC participating group, 65 (64.3 %) were SPT positive and 36 (35.6 %) were SPT negative. 50 (49.5 %) of the participants showed early reaction and 12 (11.9 %) showed a late reaction, whereas 39 (38.6 %) did not show any reaction (Table 3). Food specific OFC results show that wheat allergy is most prevalent, affecting at least 1.6 % (95 % CI 0.9–2.84 %) individuals in the total allergy patients, whereas in the OFC trial group the prevalence was 10.99 % (95 % CI 6.19–18.46 %), followed by the prevalence of egg allergy, that was 1.3 % (95 % CI 0.70–2.47 %) and 8.9 % (95 % CI 4.76–16.07 %) in total allergy patients and OFC group respectively (Table 4).

**Table 3:** Oral food challenge (OFC) test results

Total participants in OFC (n = 101)	Skin Prick Test (SPT) results N (%)	Early reaction N (%)	Late reaction N (%)	No reaction (OFC negative) N (%)
Positive	65 (64.3)	50 (49.5)	10 (9.9)	5 (4.9)
Negative	36 (35.6)	Nil	2 (1.9)	34 (33.6)
Total		50 (49.5)	12 (11.8)	39 (38.6)

**Table 4:** Food type specific OFC test results

Food type	Total patients n = 689		OFC participants n = 101	
	N (%)	95 % CI (%)	N (%)	95 % CI (%)
Animal proteins				
Milk	4 (0.58)	0.23–1.48	4 (3.96)	1.55–9.74
Beef	6 (0.87)	0.40–1.89	6 (5.94)	2.75–12.36
Mutton	2 (0.29)	0.08–1.05	2 (1.98)	0.54–6.93
Fish	5 (0.73)	0.31–1.69	5 (4.95)	2.13–11.07
Chicken	7 (1.01)	0.50–2.09	7 (6.93)	3.40–13.62
Egg	9 (1.31)	0.70–2.47	9 (8.91)	4.76–16.07
Plant foods				
Banana	Nil	Nil	Nil	Nil
Lentils	Nil	Nil	Nil	Nil
Peanut	5 (0.73)	0.31–1.69	5 (4.95)	2.13–11.07
Soya beans	Nil	Nil	Nil	Nil
Rice	6 (0.87)	0.40–1.89	6 (5.94)	2.75–12.36
Corn	7 (1.02)	0.50–2.09	7 (6.93)	3.40–13.62
Wheat	11 (1.6)	0.90–2.84	11 (10.89)	6.19–18.46

A moderate correlation (r = 0.41) was observed between food allergens’ sensitized and OFC confirmed allergy groups. But there was no positive linear relationship observed between allergy symptoms, food allergens’ sensitized, and OFC confirmed allergy groups.

## Discussion

Among 689 patients suffering from allergy diseases, sensitization to food allergens was found in 270 (39.2 %) at 95 % CI. Previously, only one local study is available reporting SPT data from the National Institute of Health (NIH) Islamabad. The prevalence of food sensitization in this report was estimated (only 2.3–3 %) in allergy patients against five selected foods (beef, mutton, egg, rice and fish), while worldwide studies reported 32–35 and 41.7 % sensitization to various food allergens in allergy patients from India (Sai et al. 2006) and Hungary (Bakos et al. 2006), respectively are comparable to our findings.

Animal proteins tested for allergy in the target population in our research included milk, beef, mutton, fish, chicken and egg. Global sensitization to beef allergens lies between 2 and 31.8 % (Orhan et al. 2009; Lessof et al. 1980; Gendeh et al. 2000; Sampson and McCaskill 1985; Burks 1999; Fiocchi et al. 1995) with a mean percentage of 13.5 %, which is consistent with findings in the present study (15.82 %). Allergy patients’ group participating in this research showed high percent sensitization to milk allergens (20.03 %). Kumar et al. (2011) in 2011 reported 21.9 % children (6 months to 6 years) with high serum IgE against milk allergens. Most of the studies focusing on milk allergen sensitization using SPT, target various age groups of children. Worldwide SPT data are highly variable, 1 % milk sensitivity was reported from Turkish hospital (Kucukosmanoglu et al. 2008), 1.1 % of random school children in Turkish population (Mustafayev et al. 2013) and 0.1 % in the general population in Germany (Zuberbier et al. 2004). Studies from other countries including USA, China, Australia and Turkey report 10.5, 6.5, 5.6 and 18.1 % sensitization to milk in general population, respectively (Orhan et al. 2009; Greenhawt et al. 2009; Hu et al. 2010; Osborne et al. 2011). While in another study from Israel, children with allergy diseases (age 0–2 years) had alarmingly high serum IgE levels (Katz et al. 2010). The study also compared the frequency of occurrence in males and females and found 62 % male and 37.9 % female children sensitized to milk. 15.5 % SPT positive patients to fish allergen were recorded here. Sai (2011) reported 11.2 % sensitization to fish in all age groups in China, whereas most of the other reports targeted children, where percentage sensitization to fish allergen was found between 0–2.7 % (Hu et al. 2010; Haahtela et al. 1980; Ben-Shoshan et al. 2010; Roberts and Lack 2005; Chen et al. 2011). SPT results for chicken meat showed 16 % sensitization in the allergy patients participating in the present study. Previous studies again show variable results ranging from 6.5 % in 5–14 years of age group from Italy (Hu 2009) up to 20 % in Madrid, Spain (Caffarelli et al. 2010). Egg allergen sensitization in Pakistani population was 21.5 %. A report from an allergy clinic in Boston, USA demonstrated similar results, where 21.4 % of 1104 allergy patients had high levels of serum specific IgE for egg allergen (Kumar et al. 2011).

Peanuts, soya beans and lentils belong to legumes that are consumed commonly in Pakistan. Sensitization to peanuts in the study group was 11.50 %. These data closely match with earlier reports from the Netherlands (Añibarro-Bausela et al. 2010) and USA (Kumar et al. 2011) where SPT results revealed 13 and 13.4 % peanut sensitization respectively. A study from the UK found 0.4 % sensitization to peanuts in children below 3 years of age. Most worldwide studies estimated low percentage sensitization to soya beans in the general population, i.e. 0–3.3 % (Zuberbier et al. 2004; Van Veen et al. 2013; Kristjansson et al. 1999; Ostblom et al. 2008; Emmett et al. 1999). One study from Hungary that carried out SPT in 20–69 years of age group, reported 8.3 % soya bean sensitization (Bakos et al. 2006), these data are comparable to the percentage (12 %) of sensitization to soya beans in the present study. For lentils (pulses) our SPT results showed 15.4 % positive reaction. A previous study from Spain found 12.6 % self-reported allergy to lentils (Dalal et al. 2002), whereas 0.04 and 2 % lentil sensitivity was reported from UK (Emmett et al. 1999) and Europe (Martinez-Gimeno et al. 2000), respectively.

Sensitization to cereals was tested here, using allergens from wheat, rice and corn. Percent sensitization to wheat (22.6 %) was the highest in the study group. Earlier reports show 0.00–13.9 % sensitization to wheat (Bakos et al. 2006; Zuberbier et al. 2004; Mills et al. 2007; Venter et al. 2008). We found 12.5 % sensitivity to rice in the study population, this is surprisingly close to a similar work from India, where 12.1 % allergy patients were SPT positive to rice (Woods et al. 2002). Another report from Malaysia found 30 % adult allergy patients sensitized to rice (Burks 1999). Sensitization to corn was higher in the study population (16 %) compared to the limited available international data (Orhan et al. 2009; Mills et al. 2007; Kumar et al. 2007; Obeng et al. 2011). In a clinic base study from the USA, 1.04 % patients self-reported allergy to corn (Obeng et al. 2011). In another investigation on randomly selected school children in Turkish population, only 0.10 % were found SPT positive for corn allergens (Orhan et al. 2009).

Forty-nine out of 689 (7.1 %) patients visiting the allergy center in this study were sensitized to banana. There is only one previous report from Egypt, where 7.5 % positive SPT to banana were recorded. Data from other regions are absent. Only 9 % patients from the allergy clinic were diagnosed of food allergy based on open OFC as opposed to 39.2 % patients that were sensitized to one or more food allergens. This is justified because all sensitized individuals may not be symptomatic.

We report 6/689 (0.87 %; 95 % CI 0.40–1.89 %) patients in the total patient population suffering from allergy to beef. Many world-wide researchers have concluded that the prevalence of beef allergy lies between 1–10 % (Lessof et al. 1980; Sampson and McCaskill 1985; Burks 1999; Fiocchi et al. 1995; Bock 1987; Werfel et al. 1997). 4 (0.58 %; 95 % CI 0.23–1.48 %) individuals in our data population showed symptoms of milk allergy during OFC. The global prevalence of milk allergy ranges between 1–3 % (Rance et al. 1999), whereas double-blind placebo controlled food challenge data show that milk allergy ranges between 0 and 3 % (Bahna 2002). Global reports on fish allergy range from 0–7.8 % in Children (Van Veen et al. 2013; Emmett et al. 1999; Werfel et al. 1997; Hochwallner et al. 2014; Soller et al. 2012; Sicherer et al. 2004; Vierk 2007; Al-Hammadi 2010; Pavlovic et al. 2014; Kajosaari 1982; Chen et al. 2012) and 0.56 % in adults (Hochwallner et al. 2014). Data in the present work shows 0.73 % (95 % CI 0.31–1.69) adults suffering from fish allergy. Chicken allergy from various regions around the world lies between 0–13 % (Sampson and McCaskill 1985; Burks 1999; Obeng et al. 2011; Lao-araya and Trakultivakorn 2012; Etesamifar 1998; Bock and Atkins 1990) whereas in our data, we calculated the prevalence of chicken meat allergy at 7(1.01 %; 95 % CI 0.50–2.09 %). Egg allergy was recorded in 9/689 individuals and calculated at 1.31 % (95 % CI 0.70–2.47 %). Worldwide reports estimated 31 % egg allergy in infants (Palmer et al. 2013) and 0–18 % of children of all ages (Hu et al. 2010; Osborne et al. 2011; Chen et al. 2011; Van Veen et al. 2013; Werfel et al. 1997; Al-Hammadi 2010; Kajosaari 1982; Sampson and Albergo 1984; Palmer et al. 2013; Osterballe et al. 2005). Data for mutton allergy is not available, however we found in the present work, that 2/689 persons did show reaction when OFC was undertaken.

No positive allergy test to soya beans and lentils was reported in this study. Allergy to soya beans in children ranges between 0–1.5 % (Van Veen et al. 2013; Kristjansson et al. 1999; Emmett et al. 1999; Obeng et al. 2011; Venter et al. 2006; Osterballe et al. 2009) whereas in adults it is 0.03 % (Vierk 2007). Lentils/legume only self-reported or clinical cross sectional data (Ostblom et al. 2008; Dalal et al. 2002; Martinez-Gimeno et al. 2000) are available but no OFC or DBPCFC reports. A case study described anaphylaxis in an 8 year old girl after ingesting lentils (Gupta et al. 2011). Such cases are rare in the population. We found 5/689 (0.73 %; 95 % CI 0.31–1.69 %) patients with peanut allergy. The prevalence of peanut allergy in school children from Montreal, Canada was 1.50 % (95 % CI 1.16–1.92 %) (Kalogeromitros et al. 1996). Researchers from different parts of the UK have reported that the prevalence of peanut allergy declines with increasing age. 1.8 % prevalence was reported in 5-year olds, 1 % of 11 year olds (Kagan et al. 2003) and only 0.8 % of 15 year olds (Pereira et al. 2005).

Wheat allergy was the most common as confirmed through open OFC test (1.6 %). Previously, Mehl in 2006 reported from Germany that 13.47 % children aged 3 months to 14 years were suffering from wheat allergy. In Pakistan we found that only 0.87 % people were suffering from rice allergy, whereas in Japanese adult atopic dermatitis patients, 2.5 % were diagnosed with a rice allergy (Hourihane et al. 2007) and 3.63 % patients in India were confirmed of rice allergy (Woods et al. 2002). Food challenge test results from different countries give variable prevalence to corn allergy. In the USA 0.2 % (Obeng et al. 2011), UK 0.1 % (Mills et al. 2007) while in Turkey it was 0 % (Orhan et al. 2009). In our data corn allergy was present in 1.02 % individuals. Although 0.1 % (n = 969) banana allergy has been reported from UK (Osterballe et al. 2005), 0.9–1.2 % (n = 324) in infants from Iceland and Sweden respectively, were diagnosed of banana allergy using food challenge tests (Van Veen et al. 2013), in the present work no banana allergy was detected.

It is concluded that among the tested group of food allergens, wheat allergy is the most prevalent in the study population. Egg allergy is the second most common type of food hypersensitivity in the group, followed by Chicken, Beef and Fish, respectively.

Results from our data along with previously reported work on food allergy, lead towards a probable hypothesis, that prevalence of sensitization to a particular kind of food depends on the frequency of consumption of that food in the study population. As we have observed here, that, wheat (most commonly eaten staple diet of Pakistan) sensitivity was the highest is our study group whereas in Japanese population rice sensitization (staple food of Japan) was reported to be the most prevalent. Conversely, it was also found that sensitization does not show any correlation to allergy symptoms, which depends on the genetic predisposition and health status of individuals and thus can not be predicted through sensitivity tests.
